# Molecular Mechanisms of Oligodendrocyte Injury in Multiple Sclerosis and Experimental Autoimmune Encephalomyelitis

**DOI:** 10.3390/ijms130810647

**Published:** 2012-08-23

**Authors:** Jilpa Patel, Roumen Balabanov

**Affiliations:** Multiple Sclerosis Center, Department of Neurological Sciences, Rush University Medical Center, 1725 W. Harrison, Suite 309, Chicago, IL 60612, USA; E-Mail: jilpa_patel@rush.edu

**Keywords:** multiple sclerosis, pathology, oligodendrocytes, cell signaling, interferon regulatory factor 1, Caspase 1

## Abstract

New evidence has emerged over the last decade indicating that oligodendrocyte injury in multiple sclerosis (MS) is not a single unified phenomenon but rather a spectrum of processes ranging from massive immune destruction to a subtle cell death in the absence of significant inflammation. Experimentally, protection of oligodendrocytes against inflammatory injury results in protection against experimental autoimmune encephalitis, the animal model of multiple sclerosis. In this review, we will discuss the molecular mechanisms regulating oligodendrocyte injury and inflammatory demyelination. We draw attention to the injurious role of IFN-γ signaling in oligodendrocytes and the pro-inflammatory effect of their death. In conclusion, studying the molecular mechanisms of oligodendrocyte injury is likely to provide new perspective on the pathogenesis of MS and a rationale for cell protective therapies.

## 1. Introduction

Multiple sclerosis (MS) is a chronic inflammatory demyelinating disease of the central nervous system (CNS) [1]. It is hypothesized that MS is caused by an autoimmune reaction against the CNS myelin and the myelin-producing oligodendrocytes [2–4]. Several lines of evidence support this hypothesis: existence of peripheral immune dysregulation, activation of self-reactive immune cells, presence of immune-mediated demyelination and oligodendrocyte injury, and beneficial effect of immunotherapy [2–4]. In addition, MS-like disease, experimental autoimmune encephalomyelitis (EAE), can be induced upon immunization of laboratory animals with myelin antigens, or adoptively transferred by activated myelin-specific T cells [5]. The etiology of MS is still unknown but the disease is associated with certain environmental and hereditary risk factors. Identified environmental risk factors include vitamin D deficiency, infectious mononucleosis (an Epstein-Barr virus infection) and tobacco smoking [6]. Hereditary risk factors are related to allele polymorphisms in the major histocompatibility complex (MHC, HLA in humans) genes, as well as in genes involved in lymphocyte function [7]. The recurrence risk in relatives for MS are approximately 25% for monozygotic twins and around 3%–5% for other siblings, which suggests a significant hereditary component [8]. Collectively, MS is conceptualized as a complex disease in which hereditary and environmental factors give rise to organ-specific autoimmunity.

Oligodendrocytes are glial cells involved in the production of CNS myelin [9]. Myelin is a lipoprotein ensheathment of the axons with about 1 μm thickness, and periodic gaps, also known as nodes of Ranvier. Its main physiological function is to accelerate the conduction velocity of the axonal action potential [10]. Myelinated axons conduct action potentials by a saltatory mode (from one node to another), which is much faster compared to the linear mode of conduction of the unmyelinated axons. A single oligodendrocyte can extend processes to numerous (up to 50) axons, and has the capacity to renew its myelin sheaths three times within 24 h [11]. Oligodendrocytes exert a trophic effect on the axons and are involved in the maintenance of their structural integrity and function [12]. Demyelination compromises the saltatory propagation of the axonal action potential and causes conduction block. In MS this is a symptom-producing pathology: failure of axonal conduction results in a multitude of neurological deficits. Chronically recurrent bouts of inflammatory demyelination results in the relapsing pattern of the disease [13]. In addition, loss of myelin deprives the axons of the trophic effects of the oligodendrocytes, which predisposes them to more severe injury, physical transection and retrograde degeneration [14,15].

Currently, there is no oligodendrocyte-based treatment of MS and all the available disease-modifying agents target exclusively immune mechanisms, providing little direct protection to oligodendrocytes and their myelin sheaths. Protecting oligodendrocytes and myelin against inflammatory injury is likely to be of clinical relevance. This opinion is fostered by the recognition that the current model of inflammatory demyelination may be an oversimplification. Traditionally, oligodendrocytes and myelin are viewed as passive targets of an uncontrolled inflammatory response. However, there are a number of clinical and experimental studies indicating that oligodendrocyte injury may occur very early in the disease process, and is likely to initiate the formation of new demyelinating lesions [4,16–22]. Experimentally, protection of oligodendrocytes against injury results in protection against EAE [18–25]. The latter is related to the suggestion that oligodendrocytes participate in the neuroimmune network by producing regulatory signals that can alter the process of inflammatory demyelination [20,22]. Hypothetically, if we understand how oligodendrocytes respond to injury then protective therapeutic strategies in MS can be developed. In this review, we will discuss the role of oligodendrocytes in CNS inflammation and the molecular mechanisms of oligodendrocyte injury and inflammatory demyelination.

## 2. Oligodendrocyte Injury in MS

**Figure 1:**
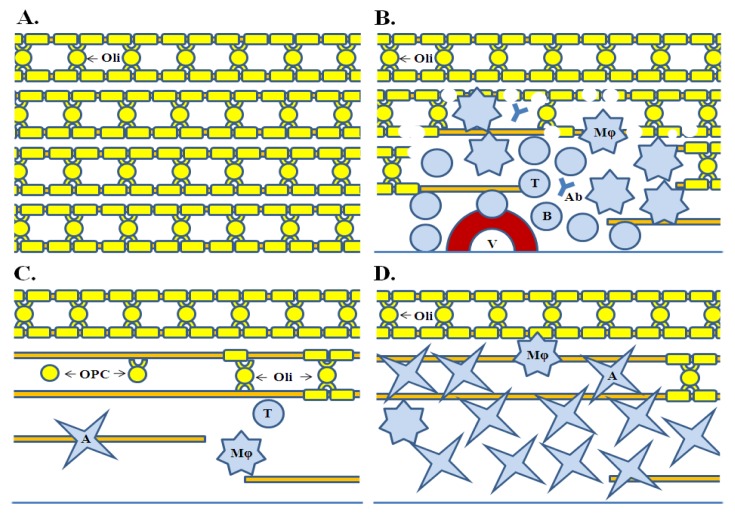
Basic pathological processes in multiple sclerosis. (**A**) Normal white matter. Oligodendrocytes and their projections form a peri-axonal (myelin) ensheathment. Lipid-rich myelin give a white-yellow appearance of the normal white matter; (**B**) Inflammation of white matter. Inflammation is associated with multifocal extravasation of immune cells (T and B cells, and monocytes/macrophages) and effector molecules (antibodies and complement). They infiltrate the tissue, damage myelin, and cause oligodendrocyte, and axonal injury. Myelin and cell debris are removed by macrophages; (**C**) Remyelination. Myelin repair is associated with appearance of oligodendrocyte progenitor cells at the lesion margins and their subseqent differentiation into mature myelin-producing oligodendrocytes. Failure of remyelination is evidenced by the paucity of oligodendrocyte progenitor cells inside the lesions and their limited differentiation; (**D**) Astrogliosis. Astrocytes invade and densely populate the lesions, forming eventually multiple gliotic scars (plaques). Astrogliosis is a permament tissue alteration. A is Astrocyte; Ab is autoantibody; B is B cell; Mϕ is Macrophage; Oli is Oligodendrocyte; OPC is Oligodendrocyte progenitor cell; T is T cell, V is Blood vessel.

Oligodendrocyte injury and demyelination in MS is a stepwise process that originates in the periphery with activation of self-reactive T cells, which may be due to immune dysregulation and/or an infectious trigger [2]. These T cells recognize their antigen in the CNS, activate local cellular elements and facilitate the recruitment of secondary effector cells and molecules (Figure 1) [3]. Once set, CNS inflammation generates a multitude of specific and non-specific mechanisms of oligodendrocyte and myelin injury [26]. Oligodendrocyte cell death and disruption of myelin integrity eventually ensues, followed by tissue disintegration [2]. Direct removal of myelin from the axons is mediated by the invading macrophages (monocytes and microglia). Remyelination may take place but this process is ineffective and commonly fails to achieve significant repair [27]. Eventually, the inflammation subsides and the destroyed tissue becomes substituted by an astrogliotic scar. In essence, existence of demyelinating lesions in MS is a consequence of myelin destruction and failure of remyelination.

**Figure 2:**
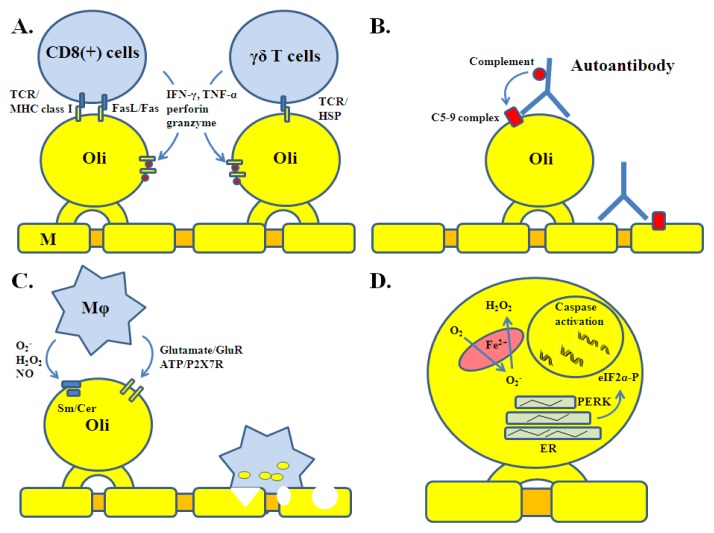
Specific and non-specific mechanisms of oligodendrocyte injury. (**A**) T cell-mediated cytotoxicity. This type of injury involves cell surface receptors, direct cell-cell interactions, and T cell-derived cytokines and molecules; (**B**) Antibody-mediated cytotoxicity. Self-reactive antibody causes activation of complement on the cell surface (C5-9 membrane complex) of oligodendrocytes, which in turn leads to pore formation and cell damage; (**C**) Macrophage-mediated cytotoxicity. Macrophages recruited to the inflammatory lesion release a number of diffusable molecules that can cause cell membrane damage and/or trigger injurious cell signaling; (**D**) Oxidative and endoplasmic reticulum stress. Intracellular metabolic perturbations lead to stress reactions and caspase activation in the abscence of a direct immune assault. ER is endoplasmic reticulum; GlutR is glutamate receptor; Mϕ is Macrophage; Oli is Oligodendrocyte; Sm/cer is sphingomyelinase/ceramide pathway.

Specific mechanisms of oligodendrocyte injury involve T and B cells (Figure 2). CD8 (+) cells are the most common lymphocyte subset identified in acute MS lesions [28]. These are MHC class I restricted T cells involved in antigen-specific cytotoxicity. CD8 (+) cytotoxicity is mediated through cell surface Fas ligand (FasL) or release of soluble molecules such as interferon-gamma (IFN-γ), tumor necrosis factor-α (TNF-α), lymphotoxin, granzyme B and perforin [29]. In settings of inflammation, oligodendrocytes upregulate the expression of MHC class I molecule (a counterpart of the T cell receptor, TCR), as well as Fas, IFN-γ and TNF-α receptors (IFN-γR, TNF-αR) rendering them direct targets of CD8 (+) cells [30–34]. Other lymphocyte types including CD4 (+) and γδ T cells are present in acute MS lesions as well [3]. γδ T cells are not restricted by MHC class molecules but they destroy oligodendrocytes expressing stress proteins such as heat-shock proteins and alpha-crystallin [35,36]. Oligodendrocytes and myelin are also targeted by antibodies produced locally by recruited B and plasma cells [4]. Together with complement, antibodies are involved in their destruction, opsonization, and subsequent phagocytosis. Myelin oligodendrocyte glycoprotein (MOG) and galactocerebroside appear to be the most important targets of antibody-mediated cytotoxicity [37,38].

Non-specific injury occurs as a complication of the inflammatory process (Figure 2). Monocytes, microglia, and astrocytes present in the lesions express receptors (MHC class II, Toll-like, and Fc receptors) and secrete a variety of molecular signals required for propagation of the inflammatory process. They also produce factors with cytotoxic activity, which contribute to the expansion of tissue injury, including proteolytic (matrix metalloproteases) and lipolytic enzymes, reactive oxygen and nitrogen species (O_2_^−^, H_2_O_2_, OH^−^, NO, ONOO^−^), and excitotoxins (glutamate, quinolonic acid) [3,4]. Oligodendrocytes are by far the most sensitive cells of the CNS to such factors. This is related to the fact that ceramide, a component of the myelin sphingolipids, can activate pro-apoptotic signaling (sphingomyelinase/ceramide pathway) in response to oxidative injury [39]. Oligodendrocyte vulnerability to oxidative stress is also related to their high metabolic rate (necessary for the myelin maintenance), large intracellular iron stores and low levels of anti-oxidative enzymes [40]. Given the large amount of their protein output, they are susceptible to endoplasmic reticulum (ER) stress, which can also activate pro-apoptotic signaling (PERK/eIF2α pathway) [41]. In addition, oligodendrocytes express glutamate (AMPA, kainite and NMDA) and ATP (P2×7) receptors rendering them susceptible to excitotoxicity [42,43]. Furthermore, locally released inflammatory cytokines such as IFN-γ and TNF-α are toxic to oligodendrocyte progenitor cells (OPC) and create a hostile environment for myelin repair [27]. The majority of these factors have overlapping molecular targets (for example cytokines and sphingomyelinase/ceramide pathway) and potentiate the processes of cell death [44]. Such high susceptibility to “bystander damage” contributes to the selectivity of myelin damage and oligodendrocyte cell loss. Chemical and enzymatic modifications of myelin lipids and proteins occur as well, producing novel immune antigens [45].

Cell death is the common fate of oligodendrocytes in the MS lesions [3,8,46]. T cell- and antibody-mediated injuries typically cause simultaneous destruction of oligodendrocytes and myelin. Some of the oligodendrocytes may survive the initial inflammatory attack despite the destruction of their myelin sheaths. With time, however, even the surviving oligodendrocytes are destroyed by the recurrent immune attacks. These cells may also die by programmed cell death since they are terminally differentiated and normally excluded from the process of remyelination [47]. Oligodendrocyte replacement from a progenitor pool does take place in some lesions as a physiological attempt at myelin repair [48]. However, this process largely fails, because of the inability of oligodendrocyte progenitor cells (OPC) to proliferate or differentiate into myelin-producing cells [27]. OPC are very sensitive to inflammatory injury (cytokine and oxidative stress)—more sensitive than mature oligodendrocytes—which limits their reparative capacity [49,50]. Changes in extracellular matrix, astrogliosis and axonal injury present further obstacles for remyelination. Nonetheless, remyelination does occur in some lesions (also known as shadow plaques) early in the disease course and in association with ongoing inflammation [51,52]. Typically, newly formed myelin is thinner and has shorter internodal segments than normal [53]. It is also very vulnerable to recurrent inflammatory injury; in fact, shadow plaques are more frequently affected by inflammatory demyelination than normal-appearing white matter [54].

Finally, there are pathological abnormalities in oligodendrocytes indicating the occurrence of additional and still poorly understood processes. Dying-back oligodendrogliopathy, yet another mechanism of demyelination, is characterized by degenerative changes in the most distal (peri-axonal) lamellae of the myelin in the presence of apparently little inflammation [4,55]. This particular mechanism may reflect the occurrence of intracellular metabolic (oxidative stress and energy failure) disturbances of oligodendrocytes early in the disease course. In corroboration, such degenerative pathology can be seen in hypoxic-ischemic disease of the white matter or in progressive multifocal leukoencephalopathy [56]. Oligodendrocyte apoptosis may occur as a primary process (prior to demyelination) in otherwise non-inflamed white matter [4,14]. In fact, oligodendrocyte apoptosis may represent the initial pathological abnormality of MS lesion formation [14]. Demyelination in the brain cortex appears to be mediated by activated microglia and in the absence of T and B cell inflammation [57]. Cortical demyelination also tends to be localized in the vicinity of meningeal inflammation and follicle-like structures [58,59].

In summary, demyelination and oligodendrocyte injury in MS are complex processes. They are secondary to an autoimmune reaction and mediated by multiple specific and non-specific (yet selective) mechanisms. Inflammatory injury progresses in a cascade-like pattern; hypothetically, the specific mechanisms first, followed by the non-specific ones. Many of the initial specific mechanisms require an active oligodendrocyte response and upregulation of certain immune relevant genes (MHC class I molecule, Fas, *etc.*). Active cell response exists even during a non-specific injury (ceramide, ER stress signaling, *etc.*). Thus, detailing the early mechanisms of oligodendrocyte injury would be of real interest since it would provide important insights into the pathogenesis of MS, and may reveal potential therapeutic targets. However, this cannot be done systematically in MS (the disease is not fatal and biopsy is rarely indicated) necessitating animal experimentation and modeling.

## 3. Oligodendrocyte Injury: Insights from Transgenic Models of EAE

EAE recapitulates many of the critical events of MS pathogenesis [5,60]. Being an inducible disease with a predictable clinical course, EAE can be used to investigate the early stages of CNS inflammation, including oligodendrocyte cell injury and death. In general, this model is an important source of information regarding the interactions of the immune system and the cells of CNS. However, traditional EAE has certain limitations since it cannot delineate oligodendrocyte response to injury or isolate its most intricate mechanisms. Experimentation with transgenic mice with genetically altered oligodendrocytes provides a new system to study these processes. Typically, these are generated by overexpression or deletion of a gene of interest using oligodendrocyte-specific expression vectors. The latter are injected into fertilized oocytes, which become integrated into genomic DNA and transmitted in mouse progeny as hereditary material [61].

**Figure 3:**
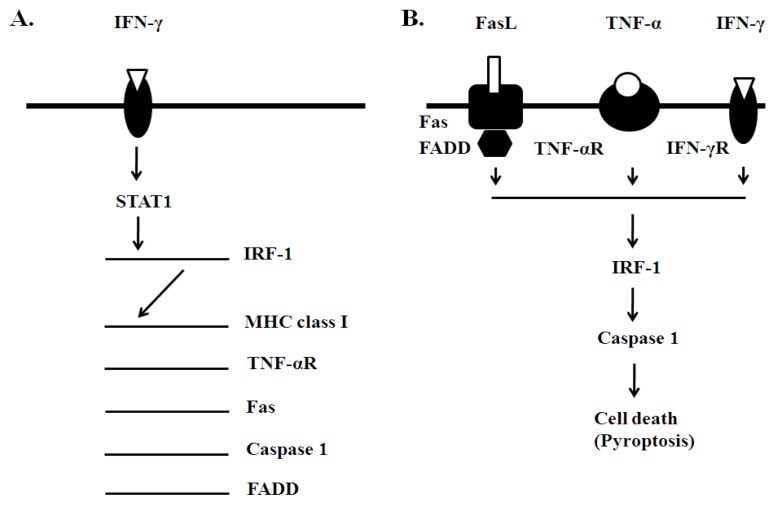
Signaling mechanisms of oligodendrocyte injury. (**A**) IFN-γ’s STAT1/IRF-1 signaling; (**B**) T cell-mediated cytotoxicity and IRF-1/Caspase 1 signaling. IFN-γ’s STAT1/IRF-1 signaling pathway upregulates the expression of a number of immune and cell-death related genes, which in turn mediate cytotoxic cell death of oligodendrocytes. Experimental (transgenic) suppression of this particular signaling pathway (STAT1, IRF-1), its downstream gene targets (TNF-αR, Fas) and adoptive/associated functional molecules (FADD, caspases) specifically in oligodendrocytes results in protection against EAE. Involvement of Caspase 1 suggests possible occurrence of pyroptosis.

Our approach has been focused on identification of an intracellular signaling mechanism linking CNS inflammation to oligodendrocyte cell death. We chose to study the signaling pathway of IFN-γ based on our previous work indicating that two of its signaling molecules, signal transducer and activators of transcription 1 (STAT1) and interferon regulatory factor 1 (IRF-1) mediate the cytotoxic effect of this cytokine on oligodendrocyte lineage cells [62,63] (Figure 3). In addition, oligodendrocytes in MS lesions demonstrate increased expression of IFN-γ-dependent genes, including major histocompatibility (MHC) class I molecule, Fas and TNF-aR [30,31]. Importantly, IFN-γ utilizes STAT1 and IRF-1 signaling in the regulation of their expression [22,62–64]. We therefore hypothesized that blocking STAT1 and IRF-1 signaling specifically in oligodendrocytes would result in cell protection, myelin preservation and limited disease.

In an effort to examine the significance of IFN-γ’s STAT1 signaling we generated a transgenic mouse line, *PLP/SOCS1* [20,63]. In these mice, SOCS1 (suppressor of cytokine signaling 1), an inhibitor of Jak/STAT1 activation, was overexpressed in the oligodendrocytes using the proteolipid protein (PLP) gene promoter. *PLP/SOCS1* mice were phenotypically normal with the exception of the inability of their oligodendrocytes to activate STAT1 and to upregulate the expression of STAT1-depedent genes (for example MHC class I). Rather unexpectedly, *PLP/SOCS1* mice developed EAE earlier compared to the control wild-type mice [20]. However, despite the accelerated disease onset these transgenic mice had shorter disease duration and recovered faster than the control wild-type mice. Pathologically, the CNS inflammation in the *PLP/SOCS1* mice remained perivascular with little myelin damage.

In a similar experimental design, we tested the significance of IRF-1 signaling. We generated a transgenic mouse line, *CNP/dnIRF-1*, by overexpressing the dominant negative form of IRF-1 (dnIRF-1) under the transcriptional control of the oligodendrocyte-specific 2′,3′-cyclic-nucleotide 3′-phosphodiesterase (CNP) gene promoter [22]. As designed, *CNP/dnIRF-1* mice were phenotypically normal. They displayed suppressed IRF-1 signaling in oligodendrocytes (dnIRF-1 functions as a competitive inhibitor of the wild-type IRF-1). Specifically, transgenic oligodendrocytes were unable to upregulate the expression of MHC class I molecule and Caspase 1 (both genes are positively regulated by IRF-1) in the presence of IFN-γ. *CNP/dnIRF-1* mice were generated directly on C57Bl/6J background for the purpose of comparing their EAE response to our previous experiments. The major finding of this study was that *CNP/dnIRF-1* mice were protected against EAE [22]. In contrast to control wild-type mice, *CNP/dnIRF-1* mice developed minimal disease and recovered within days. The inflammatory lesions in the CNS were mainly meningeal and perivascular, causing little parenchymal infiltration. Myelin and oligodendrocytes were essentially preserved; integrity of the axons remained intact as well.

Additional studies describe the significance of TNF-αR, Fas and FADD (Fas associated death domain) in oligodendrocyte cell death (Figure 3). These molecules form a signaling complex that mediates CD8 (+) cell cytotoxicity. Their expression is controlled by IFN-γ and dependent on IRF-1 [34,64–66]. EAE experiments with TNF-αR knockout (−/−) mice and transgenic mice with oligodendrocyte-specific deletion of Fas demonstrate that they are partially protected [19]. However, complete protection against EAE is observed in mice lacking both receptors on their oligodendrocytes (double deficient mice). Similarly, transgenic mice with oligodendrocyte-specific deletion of FADD develop milder disease compared to the wild-type controls, and minimal inflammatory demyelination [21]. Suppression of death signaling further downstream at the level of caspase activation in oligodendrocytes also provides protection against EAE [18]. Baculovirus p35 caspase inhibitor is used in these experiments to target oligodendrocyte caspases.

All five transgenic experiments are very consistent in their final result: protection of oligodendrocytes against cytotoxic signaling results in protection against EAE. Protection is associated not only with preservation of oligodendrocytes and myelin but also with reduction of the overall inflammatory process. This oligodendrocyte “effect” also appears to be independent of the peripheral immune responses and reproduced in adoptive EAE experiments. Such phenomena can be explained by early involvement of oligodendrocytes in the disease process, and potentially by a pro-inflammatory effect of oligodendrocyte injury and death. Several possibilities can be considered in this regard: (1) In response to inflammatory cytokines oligodendrocytes produce chemokines that can facilitate the invasion of the myelin tracts by T cells and macrophages. The limited inflammation in the *PLP/SOCS1* mice is associated with diminished CNS CXC10 (IP10) expression. This chemokine was also detected *in vitro* upon stimulation of purified oligodendrocytes with IFN-γ [20]. (2) Oligodendrocyte death may enrich the environment with myelin antigens and “danger signals”, thereby accelerating the process of antigen presentation and autoimmune stimulation. As an inflammatory disease EAE is dependent on de novo antigen presentation in CNS, a process involving the first wave of self-reactive T cells [67]. Also indicative for this possibility are the observations made in mice with defective oligodendrocyte peroxisomes; these transgenic mice develop not only demyelination and oligodendrocyte death but also spontaneous T and B cell inflammation [68]. (3) Pro-inflammatory effect of oligodendrocyte cell death may be related to a still poorly understood form of programmed cell death called pyroptosis [69]. It is associated with Caspase 1 activation, DNA fragmentation and membrane leakage of cellular contents. The latter distinguishes pyroptosis from apoptosis, where cellular contentment remained concealed by the intact cellular membrane [69]. As mentioned above, protection of *CNP/dnIRF-1* mice against EAE is associated with suppressed Caspase 1 expression in oligodendrocytes [22]. IRF-1 regulates the expression of Caspase 1 *in vitro* and *in vivo* providing a mechanistic link between inflammatory stimulus and pyroptosis [22,62]. One can suppose that pyroptosis is a positive feed-back mechanism of augmenting CNS inflammation.

## 4. Conclusion

There is growing interest in understanding the molecular mechanisms of oligodendrocyte injury in MS. New pathological data reveal the wide spectrum of oligodendrocyte pathology and the limitations of the pre-existing knowledge. Transgenic mouse experiments further delineate the significance of oligodendrocyte responsiveness (and unresponsiveness) to injury in EAE. An early active role of oligodendrocytes in CNS inflammation emerges as a conclusion, pointing to the existence of oligodendrocyte-dependent disease mechanisms. Collectively, these findings provide a new perspective on the pathogenesis of MS and may serve as a basis for developing treatment strategies focused on oligodendrocyte and myelin protection. Cell protection may be a good approach to overcome the treatment complexities of autoimmunity and the predicament of chronic immunosuppression.
